# The Prophylaxis of Tetanus: A Summary

**Published:** 1918-01

**Authors:** 


***The Prophylaxis of Tetanus : a Summary.*** By A. T. MAC CONKEY, Bacteriologist in charge of serum laboratories, Lister Institute. Abs. from the *Brit. Med. Journ.*, Dec. 11, 1915.

The author quotes the following statments from the *Memorandum on the Treatment of Wounds in War* (July 1915) :

" The prophylactic use of tetanus antitoxin is a proceeding of well established value.

" Since, in the first two months of the war, more cases of tetanus occurred than had been anticipated either by ourselves or our Allies, it was decided to direct that a preventive dose of serum should be given to every wounded man. The results have been excellent, and in the last six months there have been only 36 cases of the disease among those who received a preventive dose of serum within twenty-four hours of being wounded.

" The preventive dose of 500 U. S. A. units should be given subcutaneously at a distance from the wound at the earliest possible moment. In severe wounds medical officers not infrequently give 1,500 units; there is no objection to this, but at the same time there is no evidence that the smaller dose is insufficient if given promptly. "

The author states that this quotation is a fair expression of English experience, and proceeds at some length to confirm it by reports from other countries at war. He notes that among 83,593 cases of war wounds reported by 16 different writers from allied and enemy countries, there was an incidence of 0.65 o/o of tetanus as against an incidence of 0.36 o/o of tetanus among the 95,000 Germans wounded in the Franco-Prussian war.

Although the actual statistics at hand with regard to prophylaxis by antitoxin are exceedingly small, they indicate an incidence of only 0.26 o/o among 1,881 known cases in which preventive injections have been administered.

More significant and reliable, however, is the informal testimony from many who have used the treatment with success during the war. The estimate of its value made before the war has been amply confirmed by the severe test of actual service.

*Prophylactic Dose.* — For uniformity, the author prefers the standard of the U. S. A. unit for measurement. He notes that the 10 cc. dose of the Pasteur Institute is the equivalent of about 600 U. S. A. units. Experience recorded by 12 foreign writers, mainly German, is based upon a dose of 800 U. S. A. units. The French use 600 U. S. A. units, and the English 500. The author concludes that a dose of from 500 to 1,000 U. S. A. units is sufficient for all but a few injuries. The English dose of units, which is the lowest, has never been thought insufficient.

*Passive Immunity.* — The duration of immunity has not been definitely established. A considerable number of experiments and of isolated cases observed, indicate that incipient tetanus may be merely deferred for a period of from one to two weeks by the use of serum, and then set in, apparently as a result of weakening immunity. One case of suspended tetanus indicates that immunity is sometimes afforded for three weeks by a dose of 1,500 U. S. A. units.

It is probable, however, that large doses do not confer a longer period of immunity than small doses, and that it is therefore better to repeat a small dose of 500 units at the end of a week and again at the end of a fortnight than to give a single large dose of 1,500 units at the start.

*Very Late Developments of Tetanus.* — Many surprising cases are reported in which tetanus develops late. In one, tetanus appeared 70 days after a wound which, on the second day, had received serum treatment. Kohler and Grundmann as well as Dudgeon, Gardner, and Bawtree (all writing in 1915) report that men may be carriers of tetanus for weeks or even months. Patients may communicate it to each other or it may develop symptoms in the carrier after a long dormant period.

Cases are cited in which late infection has been caused by felt and by the East Indian fiber known as Pengawar Djambi. It also appears that injections of staphylococci may reactivate quiescent tetanus. This has been proved in experiments upon laboratory animals. Francis (1914) injected subcutaneously, on the abdomen of 26 guinea-pigs and on the backs of 26 mice, 11 vaccine viruses, 10 of which contained B. Welchii, and all of which were contaminated with tetanus spores. All but 9 of the mice developed tetanus. The 9 which were apparently immune were used for further experiments. 45 days later 6 of them were injected with a 24-hour agar culture of staphylococcus and at the end of 66 days the same experiment was tried on the remaining 3. All of the 9 mice promptly developed fatal tetanus.

By similar experiments on guinea-pigs he showed that tetanus may lie dormant for about 4 weeks, and then be reactivated either by quinine or by staphylococci. White mice will harbor spores for four months, at the end of which time the spores may be activated by staphylococci but not by quinine.

The phenomenon thus revealed deserves very careful investigation. Unfortunately there is not much clinical information of a precise nature available. It has been ascertained that the tetanus organism is present in many wounds that do not develop tetanus. There have also been recorded several curious cases in which some slight surgical interference in the neighborhood of a wound apparently healed, has brought on late tetanus. The author quotes C. J. Bond (1915) as follows : " I have records of cases in which after all incisions and sinuses round a compound fracture involving the elbow-joint or hip or other joint had completely healed, even simple passive movement of the joint under an anesthetic has lighted up a violent reaction, the reappearance of the old sepsis and the formation of local abscesses, although no incision was made, nor any solution of skin surface was produced ". Other writers confirm this testimony. Goldscheider reports the case of a soldier who apparently caused the appearance of tetanus in a wound that had completely healed, by a long walk that he was obliged to take to regain his regiment.

*Operation.* — Nothing during the war has changed the opinion held before the war, that, in cases of tetanus, operation should be avoided. The author, in support of this opinion, outlines laboratory experiments and clinical experience, proving that by the time tetanus symptoms appear, there is already a thoroughly toxic condition of the blood. If an operation is necessary, every precaution should be taken that no free toxin remain in the blood. A *large* prophylactic injection is therefore needed. Subcutaneous injections are valueless for this purpose because of their slow rate of absorption. If the injection is intramuscular, several hours must be allowed for absorption. An intravenous injection, however, permits of almost immediate operation, and its amount in the blood remains constant for nearly sixty minutes.

In summarizing the points made above, the author urges a more careful observation and report of cases in which antitoxin fails to prevent tetanus. He appends a bibliography.

